# Do future healthcare professionals have adequate knowledge about risk factors for stress urinary incontinence in women?

**DOI:** 10.1186/s12905-020-01124-0

**Published:** 2020-11-16

**Authors:** Joanna Witkoś, Magdalena Hartman-Petrycka

**Affiliations:** 1 Faculty of Medicine and Health Science, Andrzej Frycz Modrzewski Krakow University, ul. G. Herlinga -Grudzińskiego 1, 30-705 Kraków, Poland; 2 Department of Basic Biomedical Science, School of Pharmacy with the Division of Laboratory Medicine in Sosnowiec, The Medical University of Silesia, Katowice, Poland

**Keywords:** Stress urinary incontinence, Risk factors, Medical students’ knowledge

## Abstract

**Background:**

Stress urinary incontinence worsens living conditions as far as the occupational, social, mental, physical and sexual aspects of a woman’s life. Despite its real impact on the everyday lives of millions of women around the world, this problem is still disregarded and treated only as a discomfort associated with personal hygiene. Could this be due to negligence on the part of medical personnel in this matter and perhaps this lack of knowledge and proper information intended for women with stress urinary incontinence? Implementing educational activities to increase knowledge about urinary incontinence will translate into better educated women and earlier implementation of urinary incontinence treatment in the future. To properly educate women at risk of urinary incontinence, one needs to be familiar with the condition, in particular the risk factors for its development. The purpose of the study was to evaluate the degree of knowledge of students graduating from medical faculties have regarding risk factors for stress urinary incontinence in women and assess where the students’ knowledge of this problem came from.

**Methods:**

The research involved 1581 final year students of medical faculties: nursing and midwifery (258), medicine (432), physiotherapy (402) and other medical (489). The author’s survey was used for the research. The chi^2^ test was used for analysis.

**Results:**

Students in faculties of nursing and midwifery, general medicine, physiotherapy, and other medical faculties could correctly list stress urinary incontinence risk factors in 88.8%, 81.7%, 74.4% and 51.9% of their answers respectively (*p* < 0.01). The most frequently mentioned source of knowledge about stress urinary incontinence was higher level education in 82.6%, 89.8%, 90.0% and 34.4% of the respective groups’ replies (*p* < 0.001).

**Conclusions:**

Nursing and midwifery students had the greatest theoretical knowledge of stress urinary incontinence, and lesser knowledge was found among general medicine students, while physiotherapy students and students of other medical faculties had the least theoretical knowledge about risk factors for urinary incontinence. It is advisable that more emphasis be placed on educating students about stress urinary incontinence due to their insufficient knowledge, in particular for future doctors and physiotherapists who will have direct contact with patients.

## Background

Stress urinary incontinence (SUI) occurs when coughing, sneezing, laughing, or severe physical activity increases intra-abdominal pressure accompanied by involuntary urinary leakage. SUI is the most common form of urinary incontinence and might appear at any age. Patients involuntarily pass small amounts of urine, with no feeling of pressure. A characteristic feature of this type of urinary incontinence is that the symptoms disappear during night rest and that the frequency of incontinence during the day does not change. There are three degrees of intensity for stress urinary incontinence. The first degree is the lightest form of urinary leakage which occurs when there is a significant and sudden increase of pressure inside the abdomen, e.g. when coughing. The second degree is characterized by leakage of urine at moderately elevated intra-abdominal pressure, e.g. when jumping, climbing the stairs or during light physical work. The third degree is the heaviest form when involuntary urinary leakage occurs almost continuously, even when lying, standing or walking [1–6].

The epidemiology of stress urinary incontinence is not clearly established. When analysing research conducted by other authors, Serrati et al. [2] stated that it is difficult to determine the actual distribution and frequency of urinary incontinence in the society, as during the research the definition of this disorder is not strictly adhered to, and the studies are not only based on different criteria, but are also conducted in different groups of women. However, in the authors’ opinion [2] based on their analysis, the prevalence of any form of urinary incontinence ranges between 25 and 27.6%; stress urinary incontinence is considered to be the most common form of that disorder.

SUI is an interdisciplinary issue and should be considered in terms of many aspects that have a significant impact on all areas of a patient's life and on its quality. SUI is not only a problem of adult women, but a common phenomenon which occurs in different age and ethnic groups and in different clinical conditions. The etiopathogenesis of this disorder is multifactorial and complex and may result from illnesses and dysfunctions of differing organs and systems. Therefore, urinary incontinence is treated as a symptom and not as a homogenous disease entity as far as etiopathogenesis is concerned. Urinary incontinence is a symptom or complication of many serious, often chronic, female disorders, including not only the small pelvis organs and urinary tract. This symptom may appear as a result of e.g. diseases of the nervous system, such as a brain tumor, Parkinson’s disease, multiple sclerosis, or respiratory diseases, such as chronic obstructive pulmonary disease. Urinary continence depends on normal operation of the central nervous system and peripheral nerves, anatomical and functional correctness of the bladder and urethra, as well as proper support of tissues and pelvic floor muscles. Disorders or abnormalities of any of the aforementioned structures may result in problems with urinary continence. The variety of causes of urinary incontinence makes it an interdisciplinary issue, involving specialists in the area of gynecology, urology, surgery, neurology, psychology and rehabilitation, as well as primary healthcare [1, 2, 4, 5, 7–10].

Stress urinary incontinence worsens living conditions as far as the occupational, social, mental, physical [3] and sexual aspects [11] of a woman’s life. However, despite its real impact on the everyday lives of millions of women around the world, this problem is still often disregarded and treated only as a discomfort associated with personal hygiene. This lack of knowledge of SUI among medical personnel leads to a lack of how to effectively treat this problem. Why is stress urinary incontinence not discussed as much as other modern civilizational diseases? The authors wanted to find the reasons why the problem of SUI had been carried in silence by millions of women, and whether a lack of knowledge on the part of medical personnel about this disease could have led to a lack of communication with patients. Women often do not seek medical help because they are unaware that there is a possibility of SUI treatment. Could this be due to negligence on the part of medical personnel in this matter and perhaps this lack of knowledge and proper information intended for women with SUI has led to millions of women around the world to not undertake treatment of this affliction and worst yet are left to suffer alone in silence for sometimes even decades. Hendrix et al. [12] stated, the main barrier to effective management of urinary incontinence is not a lack of treatment options but rather a lack of communication between patients and their health care providers about this problem.

Considering SUI issues in literature, to the best of our knowledge, it is concluded that medical personnel’s knowledge of this condition has not yet been assessed, therefore the aim of the study was to evaluate the degree of knowledge of students graduating from medical faculties regarding the risk factors of stress incontinence in women. The authors tried to determine whether future medical staff or the youngest medical personnel currently entering the world of contact with patients, including women suffering from SUI, have sufficient knowledge to discuss SUI with patients and if so, where they obtained this knowledge. The authors wanted to determine if young people who are graduating from medical science are able to identify risk factors for this condition and if they will therefore be able to help women with whom they will have contact and be able to minimalize the risk of this condition or modify the lifestyle of affected women so that the condition does not worsen. Moreover, the authors wanted to call the attention of future medical personnel to this serious and crucial problem of SUI and encourage them to broaden their knowledge about this symptom, as well as instill in them a willingness to promote health education of SUI among their future patients.

## Methods

The study included 1581 participants, students in their final year of medical studies from two universities. The study involved 1255 women and 326 men. Group M (medicine) consisted of 432 students from the faculty of medicine, including 282 women and 150 men aged 24.9 ± 1 years. Group P (physiotherapy)—402 physiotherapy students, including 288 women and 114 men aged 25.4 ± 4 years. Group NM (nursing and midwifery)—female students of nursing and midwifery, 258 women aged 30.1 ± 8 years. Group OM (other medical)—students of other medical faculties: dental, pharmacy, medical analytics, cosmetology and public health—489 people, including 427 women and 62 men aged 24.8 ± 4 years were considered as a control group. The research was conducted before obligatory lectures at the University, and only once at each year, to prevent a possibility of students learning additional information resulting in misrepresentation of their actual knowledge. All students representing the last year of all university courses listed above participated in the study. Only people who due to their personal situation could not participate in classes scheduled on the study day did not complete the survey.

The author’s questionnaire was used in the research. A number of questions in the questionnaire were concerned with various issues related to SUI. The questions that generated the results on this study is provided as Additional file 1. In the study, a respondent was asked to list risk factors of SUI in women. It was also taken into account that the student being surveyed may not be knowledgeable of this subject, which is why the questionnaire had the option of choosing “I do not know”.

To assess where students had received information about SUI, six response options were given, indicating possible sources of their knowledge about this problem and they were instructed to choose all answers which were applicable. The options were: Higher education studies, textbooks, the Internet, patients with this condition in the family, friends suffering from SUI, a personal problem with urinary incontinence. Students could also enter additional sources of knowledge from outside the list. They were also asked to list the names of the subjects during their studies in which the topic of stress urinary incontinence was raised.

Excel 2016 and Statistica 13.0 software were used for archiving and statistical analysis. The chi^2^ test was used for analysis. Differences at a level of *p* < 0.05 were considered statistically significant.

## Results

**Fig. 1:**
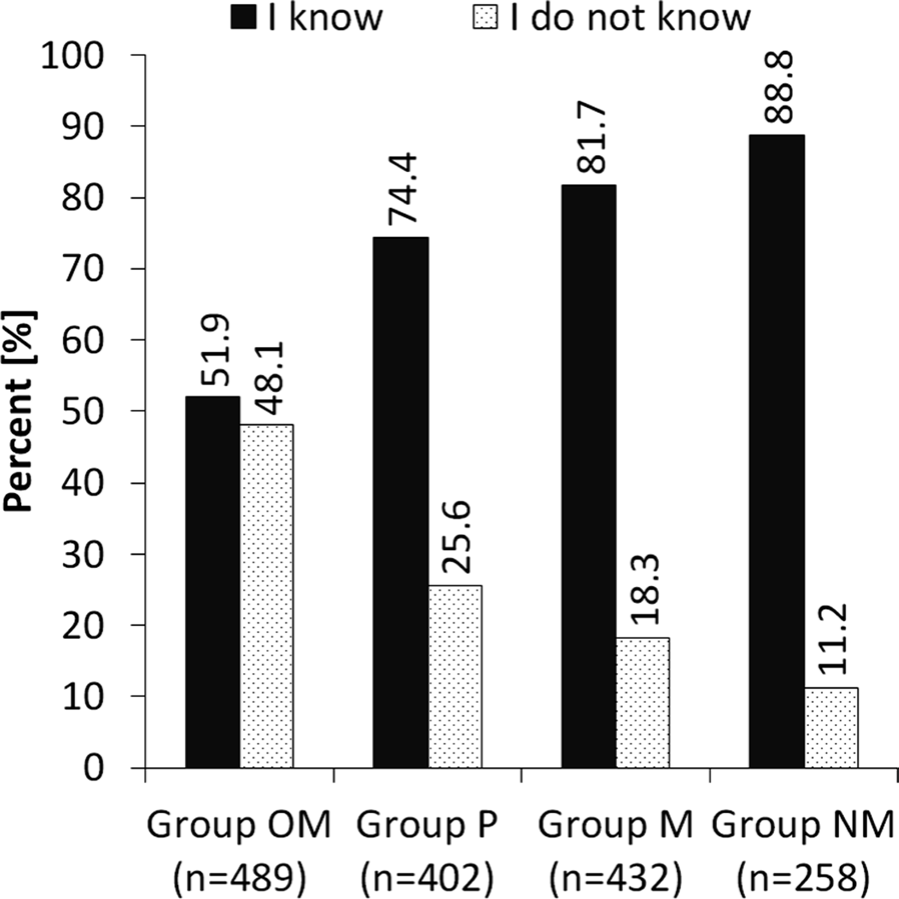
The percentage of responses ‘I know’ and ‘I do not know’ risk factors of SUI

The highest percentage of respondents who mentioned risk factors of this disease was recorded in group NM—89%, followed by group M—approximately 82%, group P—approximately 74%, and group OM—approximately 60% (Fig. 1). The statistical analysis showed significant differences between all groups (at least *p* < 0.01).

The only responses which were subject to further analysis belonged to those respondents who had listed risk factors of stress urinary incontinence. In each group, the number of respondents (n) and the average number of indications per person (i) were as follows: NM: n = 229, i = 3.2; M: n = 353, i = 3.2; P: n = 299, i = 2.9; and OM: n = 254 and i = 2.6.

**Fig. 2:**
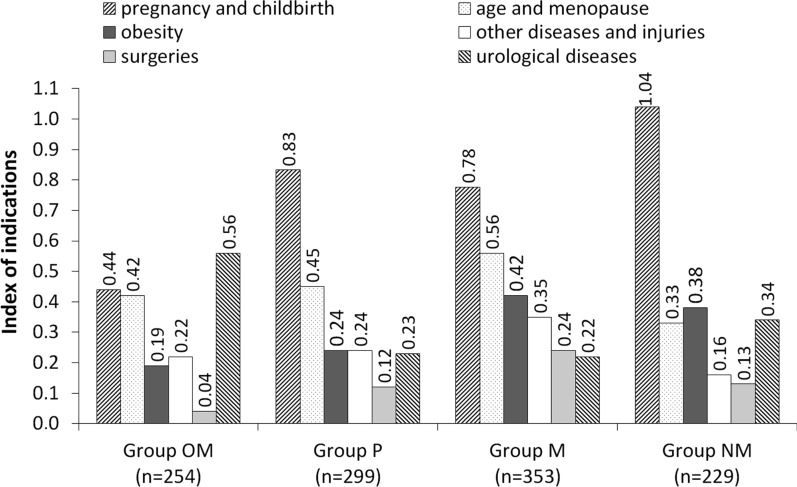
Index of indications of the six most-mentioned risk factors of stress urinary incontinence in women

**Fig. 3:**
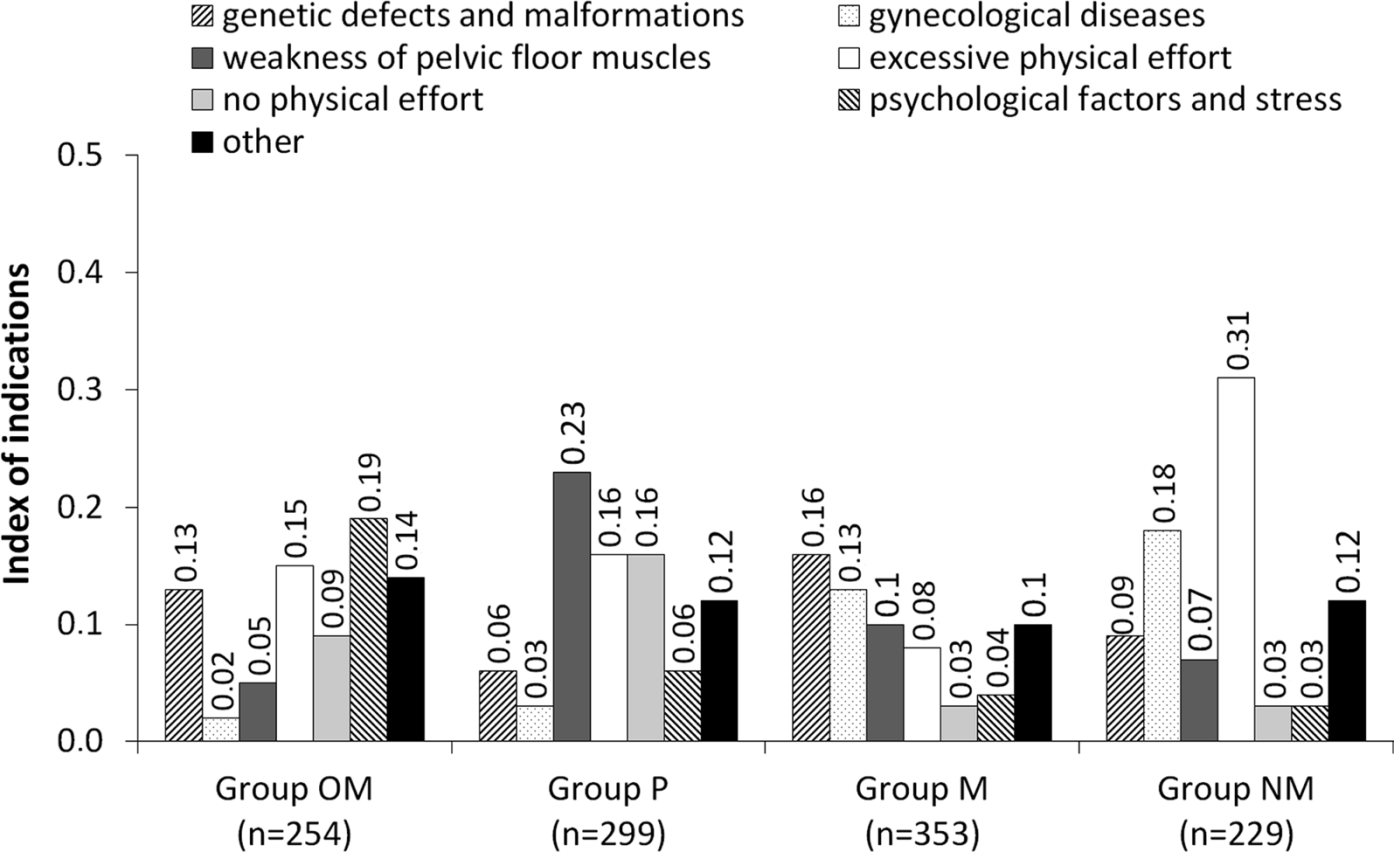
Index of indications of the seven least frequently mentioned risk factors of stress urinary incontinence in women

The risk factors mentioned by respondents were divided into thirteen categories: (1) pregnancy and childbirth, and the consequences thereof (apart from pregnancy and childbirth respondents in this category also mentioned: multiple births, high birth weight of child, numerous childbirths, pregnancy pathology, perinatal complications, no episiotomy, muscle injury during childbirth, lack of Kegel exercises, and even cesarean section and abortion), (2) age and menopause, (3) obesity, (4) other diseases and injuries (bronchial asthma, diabetes, hypertension, kidney disease, hernia, chronic constipation, cancer, water retention in the body, degenerative changes, neurological diseases, spinal cord injuries, pelvic injuries), (5) surgeries (gynecological and urological operations, abdominal surgeries, pelvic floor operations), (6) urological diseases (diseases and infections of the genitourinary system, long-lasting catheterization, weakening of urethra sphincter muscles) (Fig. 2), (7) genetic defects and malformations (developmental anomalies, anatomical changes, defects of urethra and pelvic floor muscles), (8) gynecological diseases (inflammatory conditions of the birth canal, hormone disorders, birth canal prolapse), (9) weakness of pelvic floor muscles, (10) excessive physical effort (hard physical work, professional sport), (11) no physical effort (no physical activity), (12) psychological factors and stress, (13) other (drugs and medicine, female sex, chronic cough, sneezing, too light clothing, unhygienic lifestyle, neglected personal hygiene, prolonged retention of urine, drinking large amount of fluids, poor diet, poor economic conditions, lack of knowledge about this problem) (Fig. 3).

On the basis of the obtained results, the index of indications was calculated, which was the proportion of indications in a given category of risk factors to the number of individuals who made the indications in the group. In groups NM, P and M, the highest index of indications regarding risk factors of stress urinary incontinence in women was in the category ‘pregnancy and childbirth, and the consequences thereof’ (Fig. 2) and amounted to 1.04; 0.83; 0.78 respectively. In group OM, the index for this category was 0.44. Another risk factor indicated by respondents was ‘age and menopause’ and the index of indications were: group M—0.56, group P—0.45, group OM—0.42 and group NM—0.33. The index of indications for the category ‘obesity’ in group M amounted to 0.42, in group NM to 0.38, in group P to 0.24, and in group OM 0.19. In group M, respondents indicated the category ‘other diseases and injuries’ more often than in other groups, and the index of indications amounted to 0.35, while in group P it was 0.24, in group OM—0.22, and in group NM—0.16. A similar situation was recorded in the category ‘surgeries’, which was the most frequently indicated by group M at 0.24. The remaining three groups amounted to 0.13 in group NM, 0.12 in group P, and 0.04 in group OM. With regard to ‘urological diseases’ the largest index of indications was recorded in group OM—0.56, in the other groups the index amounted to 0.34 in group NM, 0.23 in group P, and 0.22 in group M.

The index of indications of ‘genetic defects and malformations’ as a risk factor of stress urinary incontinence amounted to 0.16 in group M, 0.13 in group OM, 0.09 in group NM, and 0.06 in group P (Fig. 3). As far as ‘gynecological diseases’ is concerned, the highest index was recorded in group NM at 0.18, whereas in group M it was 0.13, in group P 0.03, and in group OM—0.02. The index regarding ‘weakness of pelvic floor muscles’ was 0.23 in group P and was higher than in the remaining groups: groups M, NM and OM respectively scored 0.10, 0.07 and 0.05. In group NM the index of indications regarding ‘excessive physical exercise’ was higher than in the other groups and amounted to 0.31. In group OM it was 0.15, in group P 0.16, and in group M—0.08. A similar index of indications was recorded in group P as previously for ‘no physical effort’ (0.16). The index of indications for this risk factor in other groups was 0.09 in group OM, and 0.03 in groups M and NM. In group OM the participants noted that ‘psychological factors and stress’ might also belong to risk factors—the index in this group was 0.19. In the other groups, it was lower—group P—0.06, group M—0.04 and group NM—0.03. Respondents also mentioned ‘other’ risk factors that could contribute to the symptoms of stress urinary incontinence in women, the index of indications was 0.14 in group OM, 0.12 in groups P and NM, and 0.10 in group M. The statistical analysis showed that the knowledge of students from different faculties about risk factors of stress urinary incontinence was statistically significant (*p* < 0.001).

**Fig. 4:**
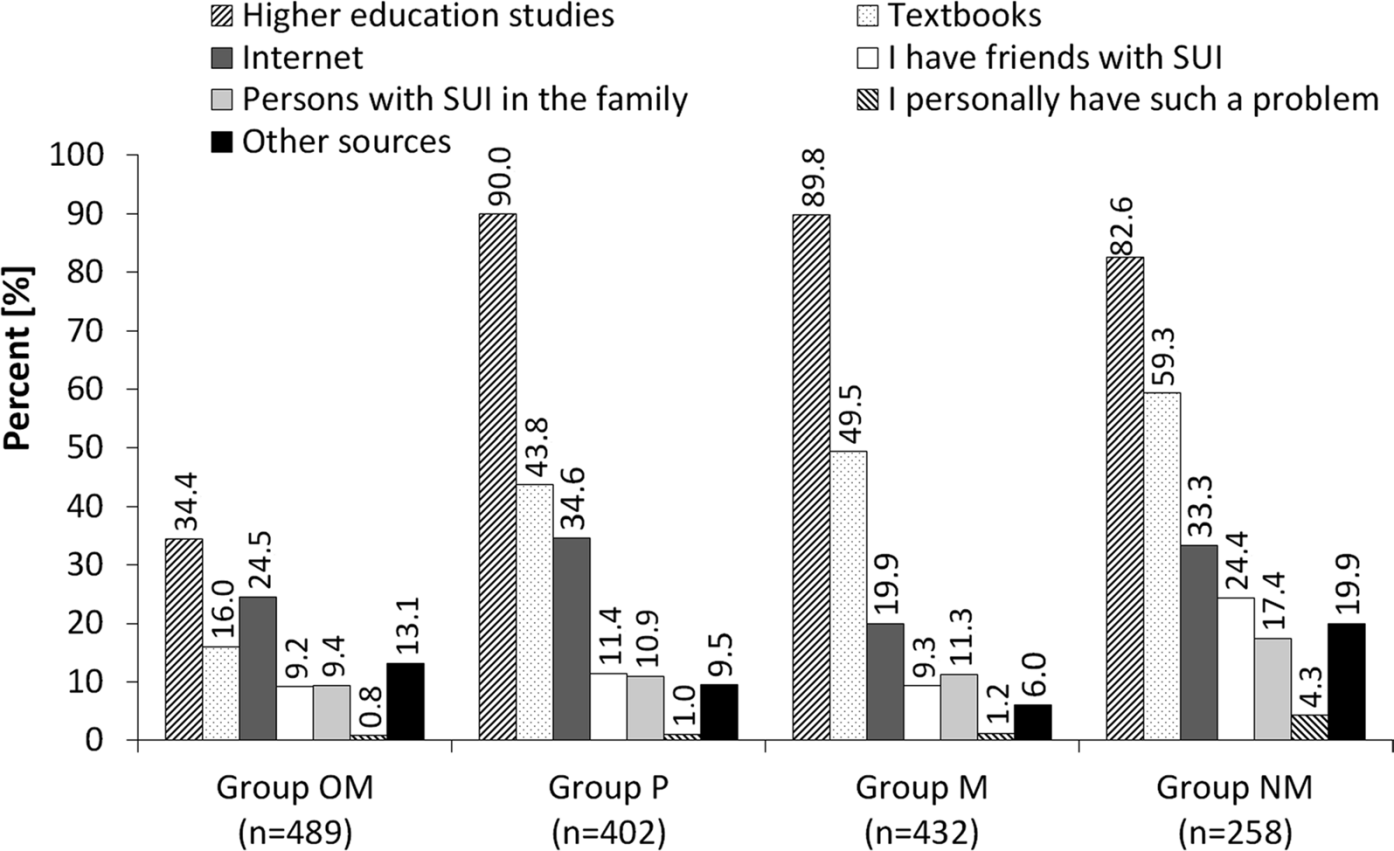
Percentage of respondents indicating different sources of acquiring knowledge about stress urinary incontinence

The most frequently indicated source of knowledge about stress urinary incontinence in all medical faculties was through their higher education studies. The percentage of students indicating such an answer was respectively: 34.4% in other medical fields, 90.0% in physiotherapy, 89.8% in general medicine and 82.6% in nursing and obstetrics. Textbooks were another important source of knowledge in the fields of P (43.8%), M (89.8%) and NM (82.6%). Figure 4 presents a detailed distribution of answers obtained on all sources of knowledge about SUI. Statistical analysis showed that the percentage of students using different sources of knowledge in the OM group was statistically significantly different from those in the P, M and NM groups (*p* < 0.001). Statistically significant differences were also noted between the NM group and the P and M groups (*p* < 0.001), as well as between the M and P groups (*p* < 0.01).

**Fig. 5:**
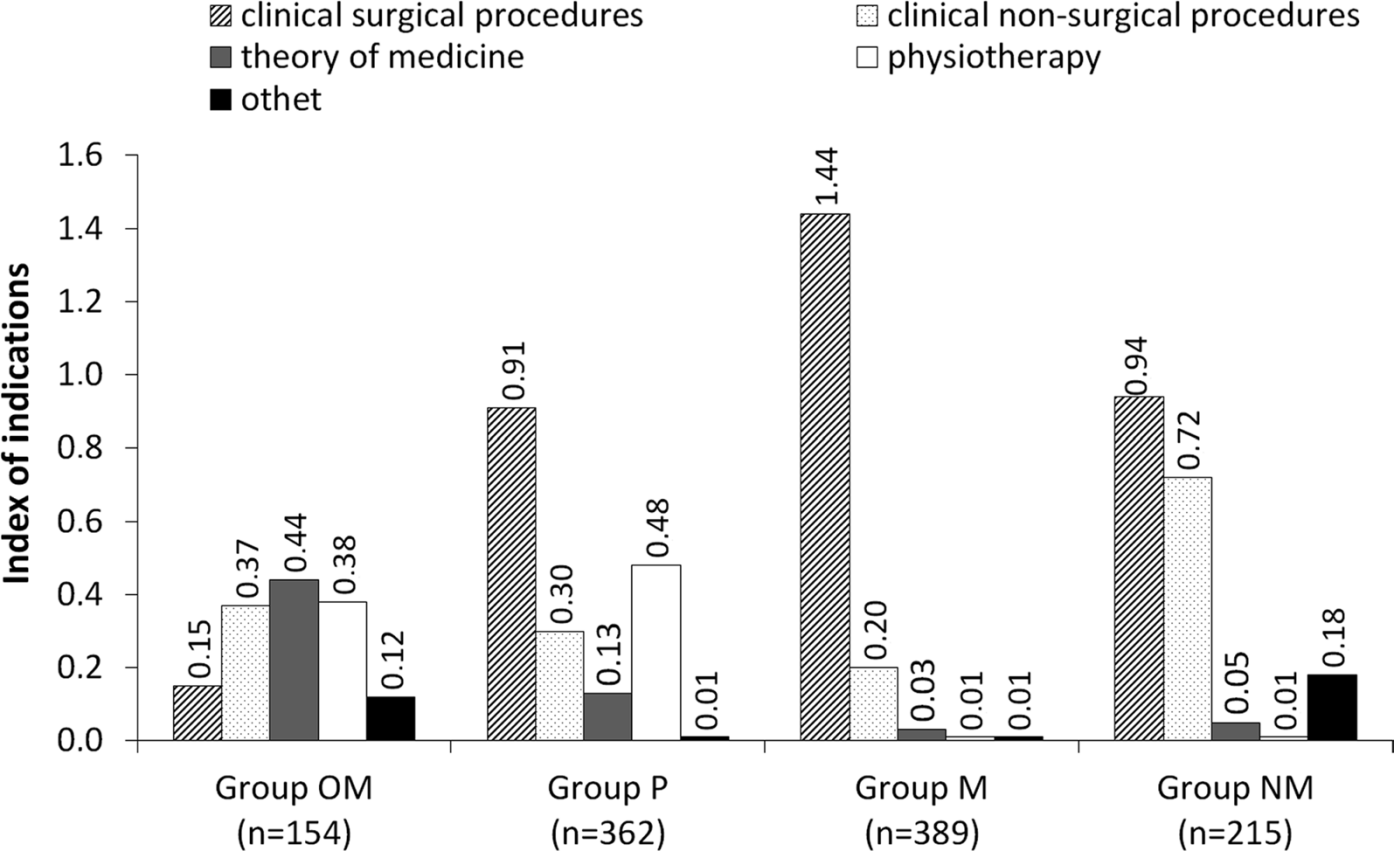
Index of indications for groups of subjects during which problems of stress urinary incontinence in women were discussed

Based on the subjects listed by the students, 5 categories were created: clinical surgical, clinical non-surgical, theory of medicine and physiotherapy, and then the index of indications was calculated, which presents the number of subjects listed in a given category per number of persons who responded in this regard. The highest index of indications concerned clinical surgical subjects which were 1.44 in group M, 0.94 in group NM and 0.91 in group P (Fig. 5). The most frequently mentioned clinical surgical subjects were surgery, gynecology and obstetrics, emergency medicine and urology. OM students most often found out about the theory of stress urinary incontinence (indications index = 0.44), e.g. through subjects of anatomy, medical biology, physiology, genetics and pathophysiology.

## Discussion

Medical personnel are expected to be familiar with SUI, particularly since it is a common and rarely discussed condition. Research sheds light on the serious and significant problems of stress urinary incontinence and draws the attention of medical personnel to this issue. It was assumed that medical staff would be interested in spreading health awareness among their future patients regarding the ways to prevent this disorder. It is advisable to put more emphasis on educating medical students regarding SUI due to their frequent and direct contact with patients suffering from this condition. Medical staff are required to have knowledge about civilization-related diseases, including SUI. Without well-educated specialist medical personnel able to see the need to act for a specific case, it is difficult to undertake long-term preventive or therapeutic initiatives. Meanwhile, knowledge of risk factors for SUI was declared by approx. 75% among the medical students surveyed (physiotherapy students) to approx. 82% medical students, which is insufficient, especially among medical staff who have direct contact with women suffering from this condition in their work.

Risk factors of SUI were divided into four groups: predisposing, causative, promoting and decompensation [13]. Predisposing factors included: genetic (women with genetic disorders such as Ehlers–Danlos syndrome, Parkinson’s disease, or diabetes have three times more increased frequency of urinary incontinence among first-degree relatives) [14–16]. Neurological changes in nerve impulse flow to the lower urinary system, e.g. [17–19]. During multiple sclerosis may be reasons of urinary incontinence, in particular of urgent and stress types [20]. Anatomical sagging and lowering of pelvic floor structures and weak urethra contraction mechanisms may also contribute to problems with urinary continence. Collagen-related pathology [21]—studies have revealed that an inborn defect of collagen structure can cause urinary incontinence as the connective tissue plays an important role in stabilizing the urethra and in maintaining proper statics of genital organs. Cultural—it was shown that the number of incidences of urinary incontinence in Western Europe and the USA (30%) is similar, but it differs from the number of people who experience urinary incontinence in Asia, where the number is lower and amounts to 20% on average. The reason of such variation has not been clearly demonstrated, but it might result from e.g. differences in child-bearing, hygiene, socioeconomic status or the use of different definitions of urinary incontinence [13]. In the conducted research, the highest index of indications regarding genetic factor of stress urinary incontinence in women was 0.16 in medical group of students. None of the student group had indicated in the vast majority neurological diseases as a SUI risk factor. These only appeared as single indications, but there were so few of them that they were classified in the group “other”. Apparently, most students were unaware of this risk factor. Students also failed to mention anatomical sagging, lowering of pelvic floor structures and defect of collagen structure. They did point out, however, “weakness of pelvic floor muscles”, and physiotherapy students indicated the such most answers, index 0.23. This is a risk factor that has already been described in 1949 by gynecologist Kegel [22], who published the results of his 15-year study using pelvic floor muscle exercises, which were used in patients with urinary incontinence, and their goal was to strengthen the weakened pelvic floor muscles. To this day, these exercises are extremely popular and are recommended by gynecologists and midwives. Currently, other methods have been introduced for strengthening this muscle group, including the especially effective electrostimulation of the pelvic floor muscles using an intravaginal electrode and impulse current with specified parameters [23].

Another group of risk factors of urinary incontinence are causative factors, which include childbirth and impairment in the pelvic muscle nerves during birth, and the development of atrophy in related pelvic floor muscles [5]—the results of most epidemiological studies suggest that pregnancy and childbirth burden the pelvic floor and thus, are an integral risk factor of urinary incontinence later in a woman’s [24–30]. Among the answers of the students graduating from medical faculties to the question regarding risk factors of stress urinary incontinence in women, the combined phrase, ‘pregnancy and childbirth, and the consequences thereof’ took the lead and was subsequently merged into a category by that name. These causative factors were definitely most often mentioned by the surveyed students, and the index of indications was the highest among nurses and midwives who most often mentioned pregnancy and childbirth, which was according to their field of study.

Hysterectomy—due to static disorder of genital organs [31]. Radiation—atrophic changes in vascularization of the lower part of the urinary system have been demonstrated which might lead to urinary incontinence and which result from pelvic radiotherapy due to e.g. cervical cancer or endometrial cancer [32]. These factors were not mentioned by the surveyed students. Obesity is a promoting factor—a close correlation between urinary incontinence and obesity was demonstrated, and it was shown that obese women with a body mass index greater than 30 kg/m^2^ experience urinary incontinence two times more frequently than women of normal weight [33–36]. A number of scientific studies highlighted that obesity is an inherent risk factor of SUI in women, but it is only one of many factors that influence the development of this condition. Therefore, it cannot be said that obese women is the sole group of women who are exposed to stress urinary incontinence. The risk of SUI in women with high BMI results mainly from an increased amount of adipose tissue, which causes pressure on the pelvic tissues and speeds their chronic tension. As a consequence, intra-abdominal pressure increases, which results in an increase of intra-bladder pressure and a reduction of the pressure difference between the urinary bladder (intra-bladder pressure) and the urethra (intraurethral pressure). This finally results in urinary incontinence. Moreover, the urethra closing mechanisms among obese women is ineffective, which may be due to chronic tension of the urogenital diaphragm [33–36]. The students surveyed were aware that obesity may contribute to urinary continence problems; the highest index of indications was noted in the group of medical students and was at 0.42.

Diet—it has been proven that certain food products such as alcohol or caffeine can increase the incidence of urinary incontinence. It is unclear whether this is related to lifestyle or the diuretic effect of these products which cause increased urinary urgency [37, 38]. Menopause—hypoestrogenism initiates atrophic changes which are manifested with mass and size reduction of genital organs and the thickness of their mucous membranes. Urogenital atrophy is also related to myofascial structures of the pelvic floor and lower part of the urinary system. As a consequence, a low level of estrogen and atrophic changes may lead to prolapse of genital organs and the appearance of urinary incontinence symptoms [39–43]. This risk factor was indicated by many students, most in the group of future doctors, indication index at 0.56 and in the group of physiotherapists, at 0.45. At this point, however, attention should be paid to literature reports [12], since a multicentre, randomized trial demonstrated that administering estrogen during menopause as hormone replacement therapy has no clinical confirmation in the treatment of stress urinary incontinence. Hendrix et al. [12] suggest re-examining the biological effect of estrogen on the lower urinary tract. The conclusion of these studies clearly shows that there is no role for estrogen in the prevention or treatment of urinary incontinence and the estrogen is no longer a treatment option for this symptom.

Occupation—a profession involving hard physical work and lifting heavy loads may lead to urinary incontinence. Physical activity level—it has been proven that both too low and too high a level of physical activity are risk factors of urinary incontinence [44–46]. The students of physiotherapy identified two completely opposite factors, namely lack of physical activity and excessive physical effort. Other groups paid almost no attention to lack of physical activity. Although many authors have shown that moderate physical exercise is associated with strengthening pelvic floor muscles and has a positive effect on reducing the risk of stress urinary incontinence, some types of physical activity may in fact contribute to these symptoms. Physical activity—such as jumping or running—often leads to urinary incontinence. Professional sport and excessive exercise may cause urinary incontinence in young women [44–46].

The surveyed students did not indicate urinary incontinence as a risk factor, as well as urinary system infections—urinary incontinence is associated with frequent infections of the urinary system. It has been demonstrated that past cystitis predisposes a mixed form of urinary incontinence. Mixed urinary incontinence is a form in which symptoms typical for stress urinary incontinence are combined with urge urinary incontinence, i.e., involuntary leakage of urine occurs, e.g., during exercise or sudden stress, combined with a feeling of sudden irrepressible urge to void. When a predominance of one group of symptoms can be established, stress urinary incontinence with detrusor instability or urge urinary incontinence with a stress component on the background is diagnosed. Risk factors for mixed urinary incontinence will include both those causing stress and urge forms of urinary incontinence, e.g., a menopausal age, pregnancies, childbirths, surgeries within the lesser pelvis, infections affecting the urinary system, or stress [1, 5, 7].

Drug-induced—drugs that cause increased urine production (diuretics) can cause urinary bladder instability, leading to urinary urgency and incontinence. Antiestrogens can cause stress urinary incontinence by reducing vascularization of the urethra and impairing the so-called ‘mucosal sealing mechanism’ [1, 2, 4, 5, 7–10]. It should be noted that among other factors mentioned by the examined students were drugs and medicine. Literature confirms that drugs, e.g. diuretics, can lead to urinary urgency, and antiestrogens to stress urinary incontinence. Smoking negatively affects synthesis of collagen by weakening pelvic floor muscles. It also induces chronic cough leading to an increase in pressure inside the abdomen, which can cause episodes of SUI. Pulmonary diseases associated with chronic cough—a connection between urinary incontinence and pulmonary diseases such as chronic obstructive pulmonary disease that causes hypoxia, night-time apnea or chronic cough resulting from, e.g. smoking. These disorders affect collagen production, which weakens the pelvic floor structure. As a consequence, when coughing, the pressure inside the abdomen suddenly increases and urinary incontinence occurs [47]. In only few individual responses, classified as “other diseases”, the surveyed students mentioned “bronchial asthma” as a risk factor for SUI. Mental illness—it has been stated that depression as a result of decreased serotonin level and decreased inhibition in the central nervous system causes urinary incontinence due to sudden urinary urgency among 60% of patients [48]. Each surveyed group of students showed a very small index of indications for mental factors that could affect the proper urinary continence; it was the highest in the other medical group and was at 0.19.

Studies have shown that the general place to gain knowledge about the studied problem were higher studies and textbooks. The subjects in which this study topic was mentioned were mostly among the groups of future doctors, nurses and midwives, and the physiotherapists mentioned clinical surgical subjects such as surgery, gynecology and obstetrics, emergency medicine and urology. In the group of students covering other medical faculties and in the group of physiotherapists, the high index of indications concerned subjects in the physiotherapy category, such as: physical therapy, kinesitherapy, clinical basics of physiotherapy or basics of physical movement education. On the other hand, considering the index of items for the category of the theory of medicine, it was found that the highest was in the group covering other medical faculties. The most frequently mentioned subjects of this group were anatomy, medical biology, physiology, genetics and pathophysiology. Approximately 10% of students of general medicine and physiotherapy, about 17% of nursing and obstetrics students and nearly 70% of students of other medical faculties said that the subject of stress urinary incontinence was not implemented in any subject during their studies. This seems likely only for the latter group. The third place among sources of knowledge was the Internet. It is certainly a quick way to find the information you are looking for and later expand upon it, and often verify it from professional sources of knowledge.

It was expected that all surveyed students would have sufficient knowledge to be able to have a professional conversation and even conduct an interview with a woman suffering from SUI. The authors of this study expected students who are studying to become physicians and physiotherapists to have more knowledge of the subject, since both of these groups belong to medical personnel who have daily and direct contact with women suffering from SUI and are obliged to know more about diseases of affluence, including SUI. It seems that the subject of SUI was well presented in medical studies but some of the students apparently did not master this issue enough to help women suffering or at risk of SUI.

The main limitation of this study was that it only included students from two universities in Poland. However, these universities are two of the largest institutions in the Silesian region educating future medical personnel. What is more, the Medical University of Silesia is one of the largest universities in Poland. At the same time, the advantage of this study is the fact that 402 graduates of physiotherapy from two different universities were examined.

Innovation of this study is its originality, because available medical databases do not include any research on knowledge of future medical personnel about stress urinary incontinence in women, which is one of the most hidden civilizational diseases that has been passed over in silence. Millions of women do not seek treatment because they are ashamed to talk about it, and medical personnel do not ask about it in their standard procedures. The article touches upon the issue of educating future medical personnel who will have direct professional contact with women who are at risk of developing urinary incontinence or are already suffering from this disorder. In addition, the article might provide inspiration for considering the importance of the disciplinary focus of a studies and the educational effects which it achieves, as well as providing inspiration for future and present medical personnel to undertake greater action for the benefit of women suffering from stress urinary incontinence. Another advantage of this article is that the questionnaire encompassed both general and very specialist knowledge, i.e. preventive measures, diagnostics and methods of conservative and surgical treatment.

## Conclusions

Nursing and midwifery students had the largest amount of theoretical knowledge about risk factors for stress urinary incontinence. A lesser knowledge was obtained by general medicine students, and lesser yet by physiotherapy students, while the least was among students of other medical faculties. It is advisable to put more emphasis on educating students about stress urinary incontinence, in particular future doctors and physiotherapists, due to their insufficient knowledge and frequent and direct contact with patients.

## Supplementary information

**Additional file 1.** The author’s questionnaire and questions that generated the results on this study.

## Data Availability

The datasets used and/or analysed during the current study are available from the corresponding author on reasonable request.

## Abbreviations

SUI: Stress urinary incontinence
Group M: Medicine
Group P: Physiotherapy
Group NM: Nursing and midwifery
Group OM: Other medical
