# Supplementary material for: Do future healthcare professionals have adequate knowledge about risk factors for stress urinary incontinence in women?
Source: BMC Womens Health. 2020 Nov 16;20:254. doi: 10.1186/s12905-020-01124-0 (PMC7670780; doi:10.1186/s12905-020-01124-0)
Supplement: Supplementary file 1 — Additional file 1. The author’s questionnaire and questions that generated the results on this study. [file 12905_2020_1124_MOESM1_ESM.pdf]

QUESTIONNAIRE

concerning stress urinary incontinence in adult women

Stress urinary incontinence (SUI) is a serious health problem in modern society. It is the last taboo in medicine and one of the most common chronic diseases more often suffered by women. This questionnaire, which we are asking you to complete anonymously, is in co-operation with the Pro-social Program 'NTM - Live Normally', which promotes awareness of the problem of urinary incontinence in Poland (www.ntm.pl). The aim of the survey is to assess medical graduates' range of knowledge relating to the area concerning stress urinary incontinence in women.

In the questionnaire, please answer all the questions by putting a cross in the appropriate column or by entering answers in accordance with your knowledge. Please DO NOT fill in the shaded fields.

1. List the risk factors associated with stress urinary incontinence in women.

| | | |
|---|---|---|
| A | | |
| B | | |
| C | | |
| D | | |
| E | | |
| F | | |
| G | I do not know | |

2. Where has your knowledge of stress urinary incontinence come from? List all the relevant sources.

| | | |
|---|---|---|
| A | Persons with SUI in the family | |
| B | I have friends with SUI | |
| C | I personally have such a problem | |

| | | |
|---|---|---|
| D | Internet | |
| E | Textbooks | |
| F | Higher education studies | |
| G | Other sources (give details) | |
| H | I have no information on this at all | |

3. During university classes, in which subjects were the problem of stress urinary incontinence in adult women discussed? List the subjects.

| | | |
|---|---|---|
| A | | |
| B | | |
| C | | |
| D | | |
| E | | |
| F | | |
| G | This topic was not discussed at all | |

4. Demographic data of the respondent.

<table>
<tr><td>Study year</td><td>Department</td><td>Course</td><td>Planned specialization</td></tr>
<tr><td></td><td></td><td></td><td></td></tr>
<tr><td colspan="2">Gender:<br>Female Male</td><td colspan="2">Year of birth:<br>…………………………………………….</td></tr>
</table>
